# PAGAL - Properties and corresponding graphics of alpha helical structures in proteins

**DOI:** 10.12688/f1000research.4952.2

**Published:** 2014-09-19

**Authors:** Sandeep Chakraborty, Basuthkar Rao, Abhaya Dandekar

**Affiliations:** 1 Plant Sciences Department, University of California, Davis, 95616, USA; 2 Department of Biological Sciences, Tata Institute of Fundamental Research, Mumbai, 400 005, India

## Abstract

Alpha helices (AH) are peptide fragments characterized by regular patterns of hydrogen bonding between the carbonyl oxygen and amino nitrogen of residues regularly spaced in sequence, resulting in spiral conformations. Their preponderance in protein structures underlines their importance. Interestingly, AHs are present in most anti-microbial peptides, although they might remain in random-coil conformations depending on the solvent dielectric. For example, the cecropin component of the chimeric anti-microbial protein designed previously by our group comprises of two AHs linked by a short stretch of random coil. These anti-microbial peptides are often amphipathic (quantified by a hydrophobic moment), aligning hydrophobic residues on one surface and charged residues on the others. In the current work, we reproduce previously described computational methods to compute the hydrophobic moment of AHs - and provide open access to the source code (PAGAL). We simultaneously generated input files for TikZ (a package for creating high resolution graphics programmatically) to obtain the Edmundson wheel and showing the direction and magnitude of the hydrophobic moment, and Pymol scripts to generate color coded protein surfaces. Additionally, we have observed an empirical structural property of AHs: the distance between the Cα atoms of the ith and (i+4)th residue is equal to the distance between the carbonyl oxygens of the ith and (i+4)th residue. We validated this using 100 non-homologous high resolution structures from the PISCES database. The source code and manual is available at http://github.com/sanchak/pagal and on http://dx.doi.org/10.5281/zenodo.11136.

## Introduction

A protein structure is formed by well ordered local segments defined by the hydrogen-bonding pattern of the peptide backbone (secondary structures), and conformations that lack any regular arrangement (random coils). The most prevalent secondary structures are alpha helices (AH) and *β* sheets, while other conformations like *π*-helix occur rarely in natural proteins ^1^. AHs are right-handed spiral conformations which have a hydrogen bond between the carbonyl oxygen (C=O) of every residue and the alpha-amino nitrogen (N-H) of the fourth residue away from the N-terminal.

DSSP is the official program used to assign secondary structure to a protein when the atomic coordinates are known ^2, 3^. Several methods can also predict an AH from the sequence ^4, 5^. Essentially, any structure prediction tool can be used to predict an AH from the sequence by first predicting the structure and then applying DSSP to the predicted structure ^6– 8^.

The niche of AHs in protein structures is widespread. AHs are the functionally significant element in several motifs (DNA binding motifs) ^9^, and the key components of any protein that permeates biological membranes ^10^. AHs are also almost always present in anti-microbial peptides (AMP) ^11^, although they may remain in random-coil conformations depending on the solvent dielectric ^12, 13^. For example, it has been recently shown that certain peptides are in random coil conformations, and achieve helical structures only by interacting with the anionic membrane model that has the same head group as the major anionic phosphatidylglycerols in bacterial membranes ^14^. For example, cecropin B, a component of a chimeric protein with anti-microbial properties that provides grapevines with enhanced resistance against the Gram-negative pathogen *Xylella fastidiosa* ^15^, is composed of two AHs connected by a small random coil ^16^. Other AMPs comprise only a single AH ^17, 18^. These peptides are characterized by a strong hydrophobic surface (defined by a hydrophobic moment ^19^), and often have charged residues, either anionic or cationic, aligned on the opposite surface ^19^. Previously, Jones *et al.* have implemented computational methods to extract the characteristics of AHs ^20^.

In the current work, we first observe and propose an empirical structural property of AHs: that the distance between the C *α* atoms of the ith and (i+4)th residue is equal to the distance between the carbonyl oxygens of the ith and (i+4)th residue. This hypothesis is validated on a set of high resolution non-homologous 100 proteins (775 AHs) taken from the PISCES database ^21^. Next, we implement the methodologies described previously ^20^ to compute the hydrophobic moments for AHs using the hydrophobicity scale used in ^22^: PAGAL - Properties and corresponding graphics of alpha helical structures in proteins. The current work is based on peptides that have solved structures which satisfy the AH property. In reality, due to conformational changes depending on solvent properties, the hydrophobic moment is not unique. There are other programs available online to do similar processing ( http://rzlab.ucr.edu/scripts/wheel/ for example). We also specify a metric associated with each helix - the ratio of the positive to the negative residues (RPNR) in the AH - which helps identify AHs with a particular kind of charge distribution on their surface. The results are outputted as the input to a graphical program TikZ (for the Edmundson wheel ^23^ and hydrophobic moment), and Pymol scripts (for showing the peptide surface). The source code and manual available at http://github.com/sanchak/pagal and on http://dx.doi.org/10.5281/zenodo.11136.

## Materials and methods

We first outline the method to obtain the coordinates of each residue in the Edmundson wheel, and the computation of the hydrophobic moment ( Algorithm 1). The input to the function is an alpha helix - either as a PDB structure or as a fasta sequence. The center of the wheel is taken as (0,0) and the radius as 5. The first residue has coordinates (0,5). Each subsequent residue is advanced by 100 degrees on the circle, as 3.6 turns of the helix makes one full circle.

To compute the hydrophobic moment, we obtain the vector by connecting the center to the coordinate of the residue and giving it a magnitude obtained from the hydrophobic scale (in our case, this scale is obtained from ^20^). These vectors are then added to obtain the final hydrophobic moment.

The results are outputted as the input to a graphical program TiKz (for the Edmundson wheel ^23^ and hydrophobic moment), and Pymol scripts (for showing the peptide surface). The protein structures have been rendered using Pymol, while the figures showing the Edmundson wheel has been obtained from TiKz. The source code is written in Perl, and made available at https://github.com/sanchak/pagal and permanently available on http://dx.doi.org/10.5281/zenodo.11136.

**Algorithm 1. Calculate hydrophobic moment**

   
**Input**: *α*H: *α* helix - either PDB or fasta sequence

   
**Input**: *TableHS*: Hydrophobic scale

   
**Output**: *TikZIN*: TikZ input file

   
**Output**: *PymolIN*: Pymol input file

   
**begin**

       Radius = 5 ; // Radius of Edmundson wheel

       initangle = 90 ; // first residue is at 12 o’clock..

       loopcnt = 0 ;

       finalvechydro = undefined ;

       centre = (0,0);

       
**foreach** *Residue _i_ in αH* **do**

             /* Find X,Y coordinate on the Edmundson wheel */

             angle = initangle - loopcnt * 100 ;

             x = Radius * cos( *val*) ;

             y = Radius * sin( *val*) ;

             thispoint = (x,y);

 

             /* Get Hydrophobic moment */

             vector = MakeVectorFrom2Points(centre,thispoint) ;

             hydrophobicvalue = GetHydrophobicScaleForResidue( *TableHS, Residue _i_*) ;

             tmpvec = normal(vector) * hydrophobicvalue ;

             finalvechydro = finalvechydro is not defined? tmpvec : finalvechydro + tmpvec;

 

             loopcnt++ ;

       
**end**

       WriteTikzScript();

       WritePymolScript();

   
**end**

## Results and discussion

### Validation of empirical property

**Figure 1.**
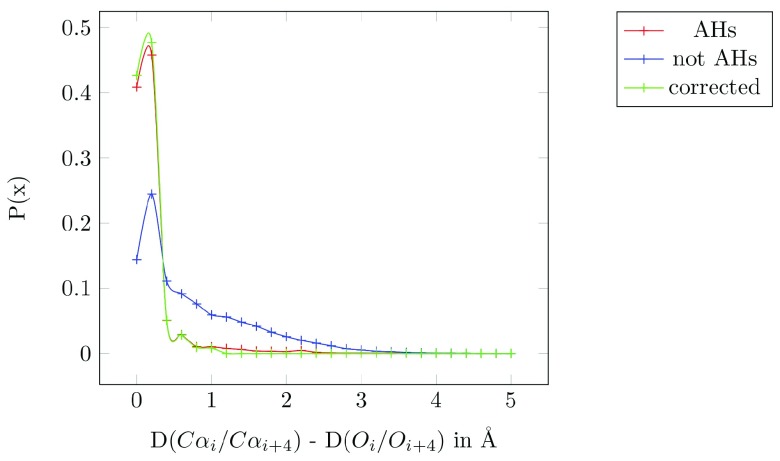
Plot of the difference between D( *Cα _i_/Cα _i_*_+4_) and D( *O _i_/O _i_*_+4_). All 775 AHs specified in the PDB files from the 100 non-homologous high resolution structures taken from the PISCES database are in red (mean=0.16 Å, standard deviation (sd)=0.34 *α* Å). All residues separated by four residues but not part of a helix are in blue (mean=0.71 Å, sd=0.75 Å). All AHs specified in the PDB files after correction are in green (mean=0.095 Å and sd=0.14 Å).

We have observed an empirical structural property that applies to the residues of any AH: the distance between the C *α* atoms of the ith and (i+4)th residue (denoted by D( *Cα _i_/Cα _i_*_+4_)) is (almost) equal to the distance between the carbonyl oxygens of the ith and (i+4)th residue (D( *O _i_/O _i_*_+4_)). We validate our hypothesis on a set of 100 high resolution, non-homologous proteins (which have 775 AHs) taken from the PISCES database ( http://dunbrack.fccc.edu/PISCES.php) ^21^. Figure 1 shows the plot of the difference between D( *Cα _i_/Cα _i_*_+4_) and D( *O _i_/O _i_*_+4_) for AHs specified in the PDB files (in red, mean=0.16 Å, standard deviation (sd)= 0.34 Å), and for all residues separated by four residues but not part of a helix (in blue, mean=0.71 Å, sd=0.75 Å).

**Figure 2.**
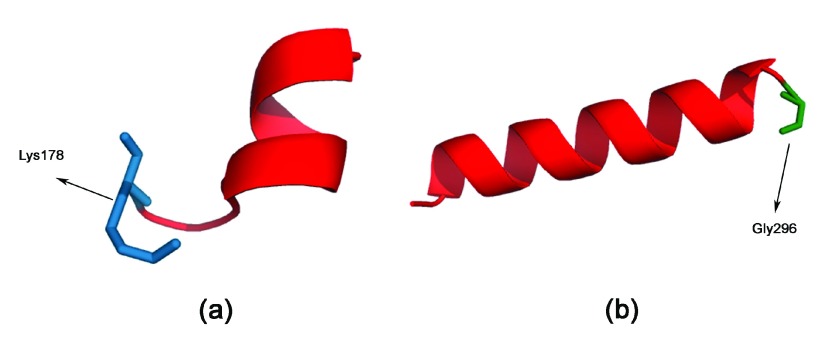
Incorrect annotations of helices in the PDB file. ( **a**) Lys178 in PDBid:1JET appears to be part of a random coil, but is annotated in the PDB file as a helix. ( **b**) Gly296 in PDBid:1ELU is mis-annotated similarly.

These results are conservative, since there are residues that are annotated as part of a helix in the PDB file which seems to be incorrect. For example, in PBD 1JET, the ninth helix spans from residues 169 to 178 - “HELIX 9 9 LYS A 169 LYS A 178 1 10”. However, the Pymol helix identification program shows part of this stretch as a random coil (Lys178 in Figure 2-a). Moreover, the distance between the carbonyl oxygen (C=O) and the alpha-amino nitrogen (N-H) of the fourth residue away from the N-terminal is 7.6 Å, which makes it improbable for them to have a hydrogen bond, the primary requisite to be part of an AH. The D( *Cα _i_/Cα _i_*_+4_) and D( *O _i_/O _i_*_+4_) for this pair is 9 Å and 8 Å, respectively: a difference of 1 Å. Even in cases where the distance between C=O and N-H is within the 3.6 Å typically required for a hydrogen bond, (PDBid: 1ELU, 12th helix), the distances D( *Cα _i_/Cα _i_*_+4_) and D( *O _i_/O _i_*_+4_) for the residue pair His292-Gly296 is 6.9 Å and 3.4 Å, respectively: a difference of 3.4 Å ( Figure 2b). In short, the helix annotation in the PDB database is often incorrect. Removing these problematic residues reduces the mean distance to 0.095 Å and the sd to 0.14 Å ( Figure 1).

There is variation in the D( *Cα _i_/Cα _i_*_+4_) even when considering the same pair of residues. For example, taking all pairs of Arg and Lys in the 775 AHs analyzed ( Table 1), we see that the values can vary from 6.5 Å in PDBid:1H16 (helix26, pair Arg583-Lys587) to 5.8 Å in PDBid:1EYH (helix5, pair Arg72-Lys76). However, as hypothesized, D( *O _i_/O _i_*_+4_) is the same as D( *Cα _i_/Cα _i_*_+4_).

**Table 1.** Occurrences of Arg-Lys pairs in 775 alpha helices found in 100 non-homologous high resolution protein structures taken from the PISCES database: RPair: Residue pair in the alpha helix with a hydrogen bond between carbonyl oxygen (C=O) and the alpha-amino nitrogen (N-H), Dhbond: Distance between carbonyl oxygen (C=O) and the alpha-amino nitrogen (N-H) of RPair, D( *Cα _i_/Cα _i_*_+4_): Distance between the C *α* atoms of RPair, D( *O _i_/O _i_*_+4_): Distance between the carbonyl oxygen of RPair, *δ*: absolute(D( *Cα _i_/Cα _i_*_+4_) - D( *O _i_/O _i_*_+4_)).

Helix	Residue Pair	Dhbond	D( *Cα _i_/Cα _i_*_+4_)	D( *O _i_/O _i_*_+4_)	*δ*
1E58.helix12	Arg188-Lys192	2.9	6.0	6.1	0.1
1H16.helix26	Arg583-Lys587	3.3	6.5	6.5	0.0
1ELK.helix4	Arg52-Lys56	2.9	6.1	6.2	0.1
1EYH.helix5	Arg72-Lys76	2.9	5.8	5.8	0.0
1F1E.helix4	Arg89-Lys93	2.9	6.1	6.1	0.0
1GXM.helix9	Arg481-Lys485	2.7	5.9	6.0	0.1
1JET.helix14	Arg290-Lys294	3.0	6.3	6.2	0.1
1EYH.helix9	Arg124-Lys128	3.0	6.2	6.2	0.0
1GCI.helix7	Arg247-Lys251	3.1	6.4	6.3	0.1
1EB6.helix3	Arg60-Lys64	3.1	6.4	6.4	0.0
1DK8.helix3	Arg140-Lys144	2.9	6.1	6.1	0.0
1GKP.helix5	Arg192-Lys196	2.9	6.1	6.1	0.0
1D5T.helix8	Arg138-Lys142	3.1	6.4	6.4	0.0

### Edmundson wheel and the hydrophobic moment

The Edmundson wheel ^23^ has been the standard way of visualizing AHs for a long time now, although there are other methods (Wenxiang diagram ^24^) to represent AHs. The Edmundson wheel shows the alignment of residues as one looks through the helix, and gives an approximate idea of the various properties of the AH. For example, a color coding differentiation of the polar and non-polar residues gives an approximation of the hydrophobic propensity of the AH. A more mathematical representation of the hydrophobic propensity is to represent each residue with a value and a sign (direction). This results in a vector representation, called the hydrophobic moment ^19^. We have chosen the hydrophobic scale from ^20^ ( Table 2), although any other hydrophobic scale could be also used. The color coding is as follows: all hydrophobic residues (positive values in Table 2) are colored red, while hydrophilic residues (negative values in Table 2) are colored in blue: dark blue for positively charged residues, medium blue for negatively charged residues and light blue for amides. We now show the PAGAL representation of a few AH peptides.

**Table 2.** Hydrophobicity scale taken from ^17^.

MET 0.975	ILE 0.913	LEU 0.852	VAL 0.811	CYS 0.689	ALA 0.607	THR 0.525	GLY 0.484	SER 0.402	HIS 0.333
PRO 0.239	PHE 1.036	TRP 0.668	TYR 0.137	GLN -0.558	ASN -0.701	GLU -1.396	LYS -1.518	ASP -1.600	ARG -2.233

**Figure 3.**
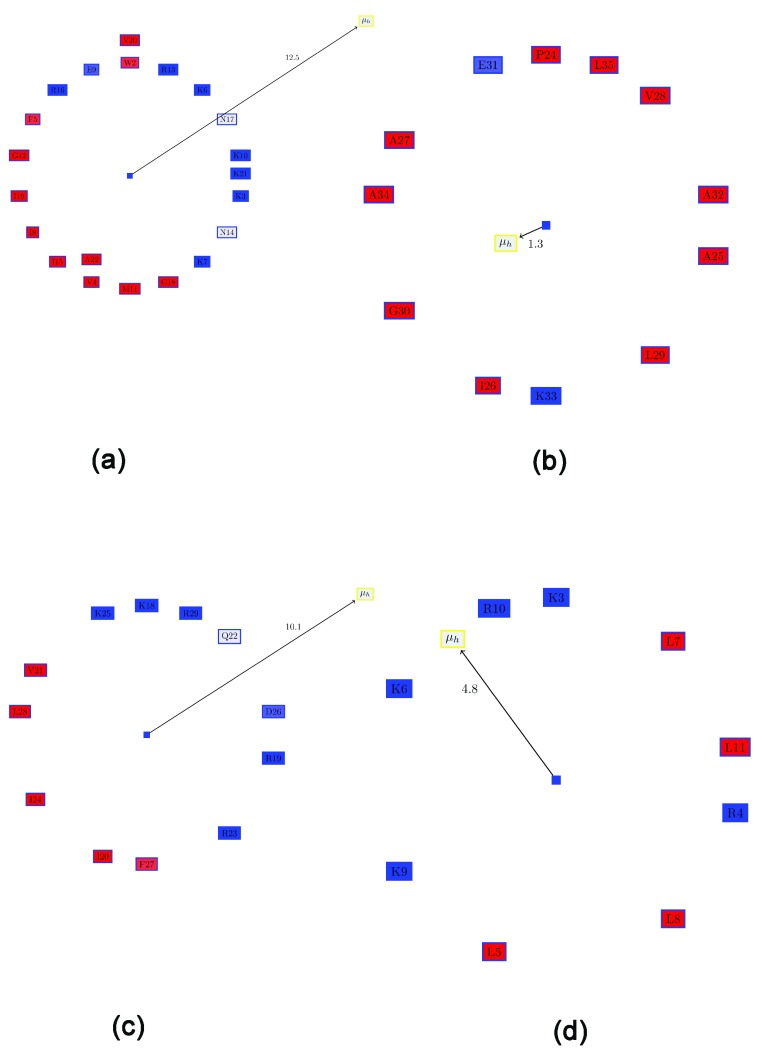
Visualizing the Edmundson wheel and hydrophobic moment of some alpha helices. All hydrophobic residues are colored in red, while hydrophilic residues are colored in blue: dark blue for positively charged residues, medium blue for negatively charged residues and light blue for amides. ( **a**) N-Terminal helix of cecropin B. ( **b**) C-Terminal helix of cecropin B. ( **c**) KR-12 peptide fragment from cathelicidin LL-37. ( **d**) *De novo* designed peptide (SP1-1) with anti-microbial activity.

**Figure 4.**
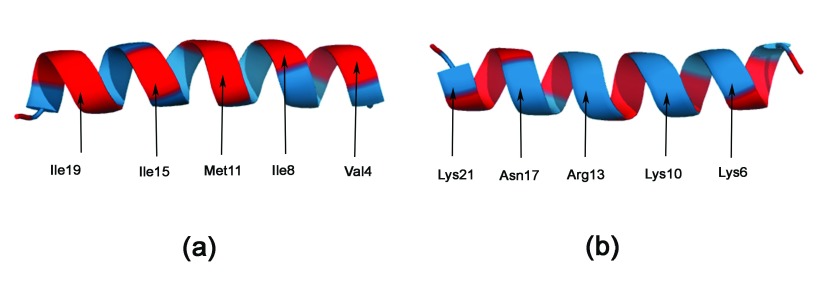
Pymol rendering of peptides showing the hydrophobic and charged surfaces for the N-terminal helix of cecropin B. All hydrophobic residues are colored in red, while hydrophilic residues are colored in blue.

***Cecropin.*** A synergistic combination of two critical immune functions, pathogen surface recognition and lysis, resulted in a chimeric protein with anti-microbial properties against the Gram-negative *Xylella fastidiosa* ^15^. The lytic domain is cecropin B, which attacks conserved lipid moieties and creates pores in the *X. fastidiosa* outer membrane ^16^. Cecropin B consists of two AHs, joined by a short stretch of random coil. Figure 3a and b shows the Edmundson wheel and hydrophobic moment of the two AHs. It can be seen that the N-Terminal AH has a large hydrophobic moment, as well as a specific positive charge distribution. The hydrophobicity of this amphipathic AH has significant bearing on the anti-microbial properties of the peptide ^25^. This can also be seen in a Pymol rendering of the peptide surface ( Figure 4). The Pymol script for this rendering is automatically generated by PAGAL. On the other hand, the C-Terminal AH comprises mostly of hydrophobic residues. Cecropin-like peptides use the synergy of these two helices - the N-terminal attaches to charged ion on the membrane, and the hydrophobic C-terminal permeates the hydrophobic inter-membrane region (known as the ‘carpet’ model ^26^).

***Cathelicidin LL-37.*** Cathelicidin LL-37 is a critical component of the innate human immune system that protects humans against infectious diseases by targeting anionic phosphatidylglycerols in the pathogenic bacterial membranes ^27^.

Recent work has demonstrated a 12-residue peptide (KR-12) corresponding to residues 18 to 29 of LL-37 is toxic to bacterial, but not human cells ^17^. Figure 3c shows the Edmundson wheel and hydrophobic moment of KR-12. The demarcation of the polar and non-polar residues is quite evident. The predominance of positively charged residues in the polar side of the peptide is also clearly visible.

***De novo designed AMPs for plant protection.*** The *de novo* design of small AMPs that inhibit plant pathogens was the focus of a recent work ^18^. One of the most promising candidates was a small peptide (SP1-1 - RKKRLKLLKRL, Figure 3d), which was “highly active against a broad spectrum of bacteria, but showed low hemolytic activity” ^18^. Although the hydrophobic moment of this peptide is much smaller than that of KR-12 ( Figure 3c), possibly due to the presence of Arg4 on the hydrophobic surface, the distribution of positively charged residues in this peptide is greater than for KR-12.

### Ratio of the positive to the negative residues (RPNR)

**Figure 5.**
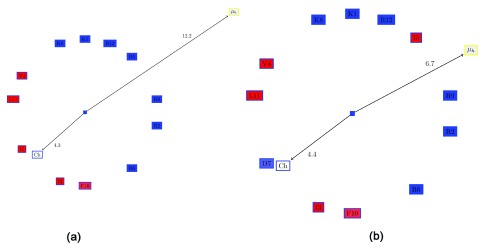
The problem in evaluating a ‘charge moment’ similar to the way the hydrophobic moment is computed. All hydrophobic residues are colored in red, while the hydrophilic residues are colored in blue: dark blue for positively charged residues, medium blue for negatively charged residues and light blue for amides. ( **a**) Edmundson wheel of a KR-12 like peptide showing the hydrophobic moment and the ‘charge moment’. ( **b**) Swapping one positive residue (R5) from the hydrophilic side with I7 and replacing it with a negative residue (D7), results in the same ‘charge moment’, although the characteristics of the helix has clearly changed.

Often, it is desirable to choose a large distribution of charged residues of a certain kind (anionic or cationic) on the hydrophilic surface. One possible method for quantifying this would be to compute a ‘charge moment’, similar to the computation of hydrophobic moments. However, such an evaluation would determine certain clearly distributions to be the same. For example, assume one semicircle of the wheel comprised only positive residues, and the other hydrophobic residues ( Figure 5a). This is a slightly modified version of KR-12 from cathelicidin LL-37. If one positive residue (R5) were moved from the hydrophilic side to the hydrophobic side (I7) and replaced with a negative residue (D7) ( Figure 5b), the ‘charge moment’ would remain the same, although the two conformations are clearly not the same. Note that the hydrophobic moment is also different, as expected. Therefore, the ‘charge moment’ is not an accurate metric. This is underlined by the fact that replacing a hydrophilic serine on the hydrophobic face with a hydrophobic residue (Ala or Val) enhanced the antimicrobial peptide activity in LL-23, a natural peptide derived from the N-terminal of LL-37 ^28^. Thus, we resort to a simple metric to allow one to choose peptides with a large proportion of charged residue of a single kind: the ratio of the positive to the negative residues (RPNR). The two peptides mentioned above will have different RPNRs: 1 ( Figure 5a) and 0.85 ( Figure 5b). Also, the current method is unable to discriminate the possible effects of substituting similar amino acids (for example replacing an arginine by a lysine). These effects are complex and difficult to computationally model, for the ‘consequences of the substitution of arginines for lysines is also modulated by the nature of the peptide into which the substitution is made’ ^14^. Such substitutions (applied to *β*-defensins also, and not AH peptides) also hold promise as future therapeutic drugs ^29^.

### Output formats

PAGAL generates a TikZ input file for drawing the Edmundson wheel and showing the hydrophobic moment (Supplementary File TikzInput.doc). TikZ is a package “for creating graphics programmatically” - http://www.texample.net/tikz/. PAGAL also generates a Pymol script to the peptide structure using the same color coding used in for the Edmundson wheel (Supplementary File PymolInput.doc).

## Software availability

### Latest source code

http://github.com/sanchak/pagal

### Source code as at the time of publication

http://github.com/F1000Research/pagal/tree/v1.0

### Archived source code as at the time of publication

http://dx.doi.org/10.5281/zenodo.11136 ^30^
