# Supplementary material for: PAGAL - Properties and corresponding graphics of alpha helical structures in proteins
Source: F1000Res. 2014 Sep 19;3:206. Originally published 2014 Aug 29. [Version 2] doi: 10.12688/f1000research.4952.2 (PMC4207245; doi:10.12688/f1000research.4952.2)
Supplement: Peer review history

# Peer review: Referee response for version 2

**DOI:** 10.5256/f1000research.5695.r6394

**Published:** 2014-10-13

**Authors:** Ramakrishnan Nagaraj

**Affiliations:** 1 Centre for Cellular and Molecular Biology, Hyderabad, India

I have read this submission. I believe that I have an appropriate level of expertise to confirm that it is of an acceptable scientific standard.

# Peer review: Referee response for version 2

**DOI:** 10.5256/f1000research.5695.r6250

**Published:** 2014-09-29

**Authors:** Guangshun Wang

**Affiliations:** 1 Department of Pathology and Microbiology, College of Medicine, University of Nebraska Medical Center, Omaha, USA

I have read this submission. I believe that I have an appropriate level of expertise to confirm that it is of an acceptable scientific standard.

# Peer review: Referee response for version 1

**DOI:** 10.5256/f1000research.5288.r6102

**Published:** 2014-09-12

**Authors:** Guangshun Wang

**Affiliations:** 1 Department of Pathology and Microbiology, College of Medicine, University of Nebraska Medical Center, Omaha, USA

The authors aimed at further define features for helices, which are key structural elements in polypeptides. Based on the traditional hydrophobic moment, they proposed the concept of charge moment. It is interesting but I am a little bit of doubtful how useful the charge moment will be. In particular, the authors found the “Swapping one positive residue (R5) from the hydrophilic side with I7 and replacing it with a negative residue (D7), results in the same ‘charge moment’” (Figure 5). In the case of helical antimicrobial peptides, such a swap may have a detrimental effect on peptide antimicrobial activity. For example, even a change of a hydrophilic residue serine on the hydrophobic face with a hydrophobic residue influenced peptide activity ( Wang G *et al.*, 2012). The authors may refine this idea and conceive the possible use of charge moment. Based on charge moment, is there any clue that interfacial charged residues of antimicrobial peptides play a larger role than non-interfacial ones in determining antimicrobial activity? Will it be possible to incorporate the observation that arginines are usually more important than lysines in determining peptide activity (see Mishra B *et al.*, 2013)?

I have read this submission. I believe that I have an appropriate level of expertise to confirm that it is of an acceptable scientific standard, however I have significant reservations, as outlined above.

# Response

**Published:** 2014-09-17

**Authors:** Sandeep Chakraborty

**Affiliations:** Tata Institute of Fundamental Research, India

We would like to thank you for taking the time and reviewing our paper. Please find our response to your suggestions below. We have incorporated the changes in the new version.

**The authors aimed at further define features for helices, which are key structural elements in polypeptides. Based on the traditional hydrophobic moment, they proposed the concept of charge moment. It is interesting but I am a little bit of doubtful how useful the charge moment will be. In particular, the authors found the “Swapping one positive residue (R5) from the hydrophilic side with I7 and replacing it with a negative residue (D7), results in the same ‘charge moment’” (Figure 5). In the case of helical antimicrobial peptides, such a swap may have a detrimental effect on peptide antimicrobial activity. For example, even a change of a hydrophilic residue serine on the hydrophobic face with a hydrophobic residue influenced peptide activity (Wang G et al., 2012). The authors may refine this idea and conceive the possible use of charge moment. Based on charge moment, is there any clue that interfacial charged residues of antimicrobial peptides play a larger role than non-interfacial ones in determining antimicrobial activity? Will it be possible to incorporate the observation that arginines are usually more important than lysines in determining peptide activity (see Mishra B et al., 2013)?**

The relevant reference you have pointed out (Wang G et al., 2012) underlines the point are making - that the charge moment (analogous to the hydrophobic moment) is not a good metric. Thus, `we resort to a simple metric to allow one to choose peptides with a large proportion of charged residue of a single kind: the ratio of the positive to the negative residues (RPNR).'. I apologize that this point has been confusing, so I have explicitly clarified this aspect. Also, the reference helps to establish our point.

Unfortunately, we would be unable to differentiate between lysines and arginines using the current methodology. However, this is a salient point that we need to discuss so that future work may address this.

We hope to have addressed your concerns with these modifications.

# Peer review: Referee response for version 1

**DOI:** 10.5256/f1000research.5288.r5988

**Published:** 2014-09-12

**Authors:** Ramakrishnan Nagaraj

**Affiliations:** 1 Centre for Cellular and Molecular Biology, Hyderabad, India

1. AHs are not invariably present in **ALL** antimicrobial peptides. The formation of AHs in antimicrobial peptides is dependent on solvent conditions. The statement to this effect should be edited appropriately.
2. The proposed empirical structural property of AHs is OK as it is validated on a set of high resolution non-homologous proteins from a database.
3. Extension of the analysis to antibacterial peptides such as cecropin and LL37 may not be valid. These peptides are unstructured in water and fold into amphipathic helical structure only in media of low dielectric constant. Also, the helical structures are not rigid as observed in protein crystal structures and show considerable conformational flexibility in solution. Hence, a unique value of hydrophobic moment will not be very meaningful.  Solvent effects need to be taken into account. This point (3) needs to be addressed and clarified.

I have read this submission. I believe that I have an appropriate level of expertise to confirm that it is of an acceptable scientific standard, however I have significant reservations, as outlined above.

# Response

**Published:** 2014-09-17

**Authors:** Sandeep Chakraborty

**Affiliations:** Tata Institute of Fundamental Research, India

We would like to thank you for taking the time and reviewing our paper, and for your helpful comments. We have modified our manuscript to reflect this changes, and hope that these are satisfactory.
